# Zinc translocation from Zn-sufficient to Zn-deficient roots as an adaptation to heterogeneous Zn availability

**DOI:** 10.1186/s12870-025-07391-z

**Published:** 2025-10-08

**Authors:** Magdalena Pypka, Diana Davydenko, Katarzyna Sowa, Julia Maksymiuk, Paweł Wróbel, Tomasz Kołodziej, Paweł Korecki, Oskar Siemianowski

**Affiliations:** 1 Faculty of Biology, Institute of Experimental Plant Biology and Biotechnology, Department of Plant Metal Homeostasis, University of Warsaw, Miecznikowa Street 1, Warsaw, 02-096 Poland; 2 National Synchrotron Radiation Centre SOLARIS, Jagiellonian University, Czerwone Maki 98, Krakow, 30-392 Poland; 3 Institute of Physics, Jagiellonian University, Łojasiewicza 11, Krakow, 30-348 Poland; 4 Faculty of Physics and Applied Computer Science, AGH University of Krakow, al. Mickiewicza 30, Krakow, 30-059 Poland

**Keywords:** Zinc transport, Soil heterogeneity, Microelements, Xylem, Phloem, Micro-XRF, Tobacco

## Abstract

Zinc is essential for plant development and human health. While the effects of soil nutrient heterogeneity on plant growth were studied for macronutrients, adaptive mechanisms for micronutrients like Zn remain largely unexplored. We investigate Zn homeostasis in *Nicotiana tabacum* plants grown in a transparent soil medium mimicking natural soil conditions with spatially heterogeneous Zn availability. Our findings suggest a previously unrecognized mechanism in which Zn is translocated between lateral roots, moving from Zn-sufficient to Zn-deficient ones, mitigating Zn deficiency responses and reducing Zn uptake. Under heterogeneous Zn conditions, the expression of key Zn homeostasis-related genes (encoding Zn importer - NtZIP4B, Zn exporter - NtHMA4a/b and Zn chelator – NtNAS) was significantly altered. NtHMA4a/b expression was influenced by the vertical positioning of the Zn-sufficient medium, while NtZIP4B and NtNAS showed suppressed expression in roots under heterogeneous conditions compared to homogeneous Zn-sufficient conditions. This suggests a systemic regulatory mechanism coordinating Zn allocation depending on whole root system Zn access. Elemental analysis revealed reduced overall Zn concentrations in plants grown in heterogeneous Zn media, with elevated Zn levels in leaf veins. This study uncovers a novel mechanism of Zn translocation within the root system in response to heterogeneous Zn supply, highlighting the complexity of micronutrient homeostasis and its adaptive regulation.

**Supplementary Information:**

The online version contains supplementary material available at 10.1186/s12870-025-07391-z.

## Introduction

It is projected that almost 582 million people will be chronically undernourished by 2030. Although the number is declining, insufficient nutrient intake/absorption still affects 6.8% (45.0 million) of children under 5 years old [1, 2]. Zinc deficiency in soils is a significant issue, potentially affecting as much as 50% of the world’s agricultural lands [3]. Zinc is a divalent transition metal and an essential micronutrient for plants, playing critical roles in numerous biological processes. Approximately 9% of the eukaryotic proteome utilizes zinc [4]. Over 300 enzymes, spanning all six major enzyme classes (oxidoreductases, transferases, hydrolases, lyases, isomerases, and ligases), require zinc as a cofactor. Furthermore, zinc serves as a crucial structural component in proteins, including alcohol dehydrogenases, protein kinases, and transcription factors (TFs) [5]. Symptoms of zinc deficiency in plants typically manifest as stunted growth and chlorosis, leading to substantial reductions in crop yields. As plants are the primary source of zinc in human diets, reduced zinc content in crops can result in zinc malnutrition, a condition that currently affects an estimated 17.3% of the global population [3, 6]. In light of the growing global population, there is an urgent need to increase agricultural productivity by 70% over the next four decades [7]. Achieving this while maintaining or improving the nutritional quality of crops presents a dual challenge, as efforts to enhance yield (primarily carbohydrate content) often compromise the accumulation of essential micronutrients, including zinc.

Plants, as sessile organisms, rely entirely on the nutrients available in their surrounding environment. However, under natural conditions, root systems frequently encounter nutrient heterogeneity in soil [8]. For instance, some roots may experience Zn deficiency, while others access Zn-sufficient zones. Plants have evolved intricate mechanisms to tightly regulate their responses to fluctuating Zn availability [9, 10]. Central to this regulation is the control of Zn transport within and between cells, tissues, and organs. This is achieved primarily by modulating the expression of Zn transporters, their localization to the plasma membrane, and their subsequent removal or recycling [10, 11]. Major families of Zn transporters include ZIPs (ZRT/IRT-like proteins), NRAMP (Natural Resistance-Associated Macrophage Protein), and YSL (Yellow Stripe-Like, Zn-ligand transport), which facilitate Zn influx into the cytoplasm. Conversely, Zn efflux is mediated by CDF/MTP (Cation Diffusion Facilitator/Metal Tolerance Protein), HMA (Heavy Metal ATPase), CAX (Cation/proton eXchanger), ZIF (Zinc-Induced Facilitator), and VIT (Vacuolar Iron Transporters) [12, 13]. Zn homeostasis is further supported by Zn chelators such as malate, citrate, histidine, and nicotianamine (NA), which prevent undesirable Zn binding and facilitate efficient transport [11].

Zn uptake occurs predominantly in its ionic form (Zn²⁺) from the soil solution via roots [14]. Once absorbed, Zn is distributed through both symplastic and apoplastic pathways [15]. In the root’s central cylinder, transporters such as NtHMA4a/b in tobacco mediate Zn efflux into the xylem, enabling root-to-shoot translocation [16, 17]. In leaves, Zn is retrieved from the xylem (apoplast) by ZIPs and NRAMPs, such as NtZIP1-like, NtZIP4, NtZIP5, NtZIP11, and NtNRAMP3 in tobacco [18]. Studies on Zn transporters have mainly been conducted under homogeneous growth conditions, where nutrients are evenly distributed in the medium (e.g., hydroponics). Under these conditions, it was shown that Zn transporters NtZIP4B and NtZIP5B are expressed mostly in the central cylinder in the middle part of the roots when Zn is sufficient and in the epidermis/cortex of most roots, including the root apical part, under Zn deficiency [9, 19].

Research on nutrient heterogeneity has largely focused on root architecture responses; for instance, Giehl (2012) demonstrated that heterogeneous Fe distribution promoted lateral root growth in Fe-rich zones to sustain shoot Fe content [20]. However, investigations into the dynamics of micronutrient distribution, particularly under heterogeneous Zn conditions, remain limited.

In this study, we grew tobacco plants in a transparent soil medium designed to mimic soil conditions [21]. This three-dimensional setup allowed the application of alternating nutrient concentrations to different regions. Compared to the split-root system, our approach allows for the creation of both vertical and horizontal Zn heterogeneity within a single root system. In contrast, the split-root system enables only vertical heterogeneity where the main roots are often cut to create two evenly sized root systems (from lateral roots) that grow in separate containers. The connection between roots is then provided through the hypocotyl only and root to root transport would be limited. Using transparent soil system, we demonstrated that Zn can be translocated between lateral roots, from Zn-sufficient to Zn-deficient regions. This surprising finding suggests the existence of previously unknown Zn homeostasis mechanisms in roots, likely involving an active and complex process mediated by Zn transporters. These mechanisms would necessitate the unloading of Zn from the xylem and its subsequent loading into the phloem, a process supported by Zn status sensing and signaling to ensure precise execution within specific root or shoot regions, independent of Zn concentration in the surrounding medium. Furthermore, we analyzed how different Zn distribution scenarios affected Zn content and distribution in shoots. This work provides new insights into plant Zn homeostasis, particularly regarding Zn translocation between tissues and organs, and may inform future agricultural practices to address Zn deficiency.

## Results

### Plants adjust Zn homeostasis when grown in Zn heterogeneous conditions

**Fig. 1:**
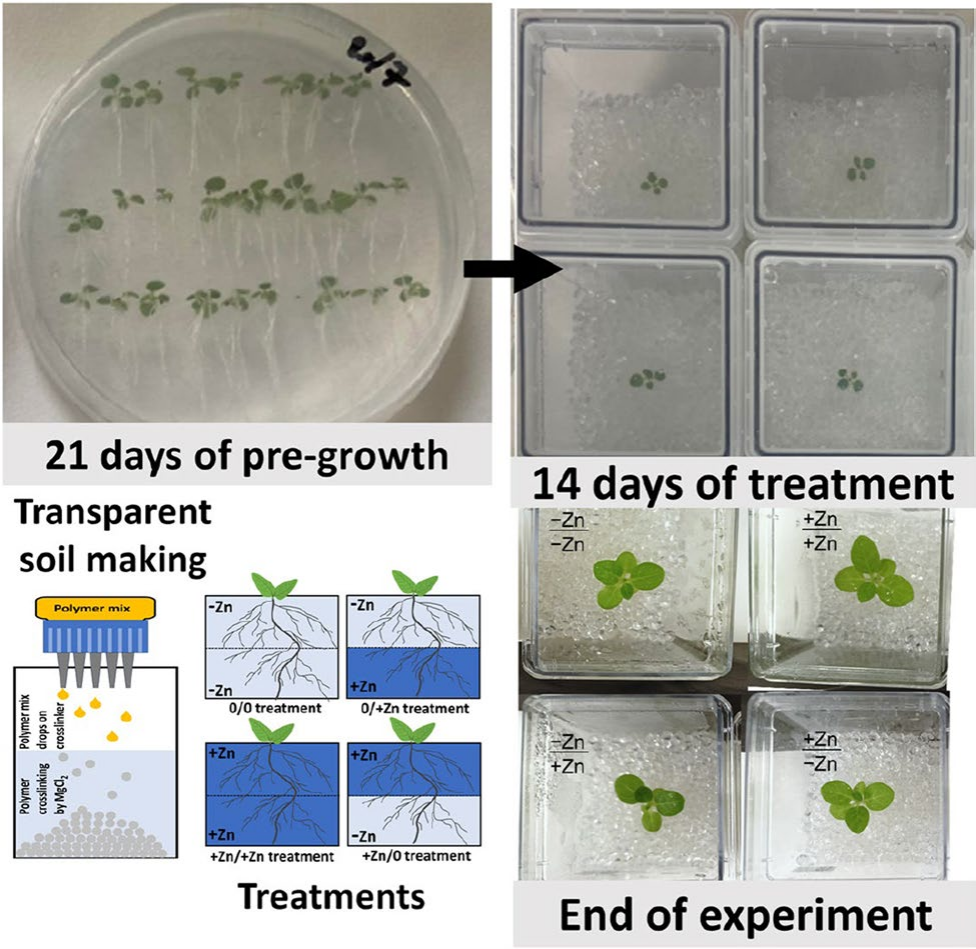
Experimental workflow illustrating plant growth and treatment setup. Plants were pre-grown for 21 days on transparent soil (top left) with quarter-strength Knop’s medium supplemented with 2% (w/v) sucrose and 1% (w/v) agar, followed by 14 days of treatment in customized growth chambers with defined conditions (top right). Transparent soil preparation and applied treatments are detailed with schematics (bottom left). Representative images at the end of the experiment (bottom right)

We were interested in identifying the Zn-related control of NtZIP4B expression (Zn importer involved in Zn uptake to cytoplasm [9, 18]) in plants grown in a medium that mimics soil conditions, including its heterogeneous Zn distribution. For this experiment, we tested tobacco expressing ß-glucuronidase (GUS) under the control of the *NtZIP4B* promoter (*promNtZIP4B::GUS*), which was selected due to its well-characterized expression pattern in tobacco [9]. Plants were grown in transparent soil [21] for 14 days under conditions where either the upper or lower half of the medium contained Zn-sufficient or Zn-deficient conditions (**-Zn**/+1 µM Zn and + 1 µM Zn/**-Zn**; Fig. 1). As controls, we included plants grown under homogeneous Zn distribution, with both halves of the medium prepared at the same Zn concentration (**-Zn**/**-Zn** and + 1 µM Zn/+1 µM Zn). Quantification was performed using the apical root segment, defined as the 2 cm region from the root tip.

**Fig. 2:**
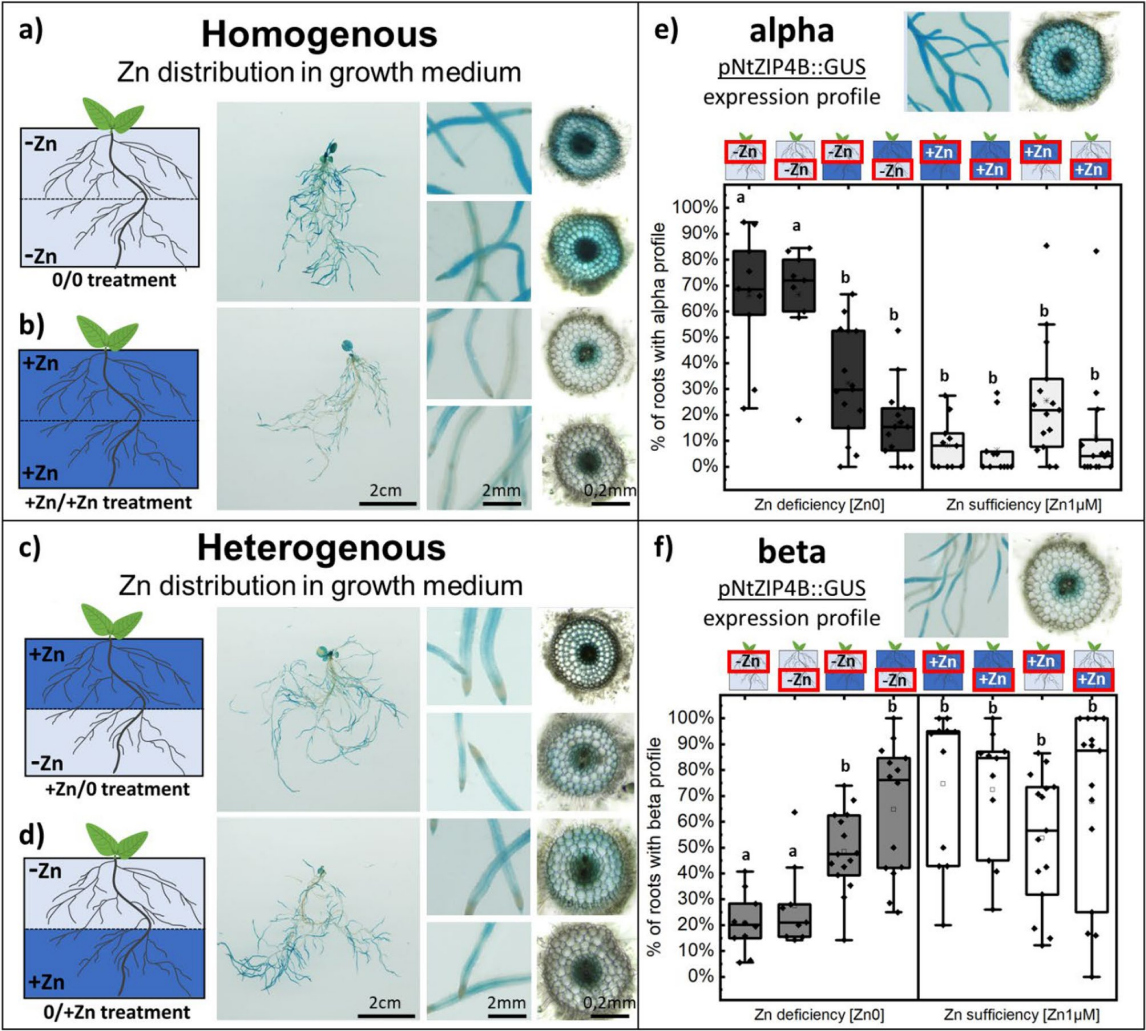
Effects of homogeneous and heterogeneous Zn distribution on root development and pNtZIP4B::GUS expression profiles in growth medium. **a–d** Homogeneous Zn conditions (**-Zn**, +Zn) and heterogeneous Zn conditions (**-Zn**/+Zn, +Zn/**-Zn**) are depicted alongside representative root images and GUS expression in roots. Insets show detailed views of root zones with varying GUS activity. **e**,** f** Quantitative analysis of pNtZIP4B::GUS expression profiles (alpha and beta) in Zn-deficient and Zn-sufficient conditions. Box plot showing data distribution with whiskers extending to the outermost points within the upper and lower inner fences (1.5 × IQR) depict the percentage of roots exhibiting specific expression profiles under different treatments. Statistical differences (pairwise t-test) between treatments are indicated (letters denote significance, *p* < 0.05). Black rhomboid shows data point. Pictograms above the graph indicate the region of the medium where samples were taken (red rectangles) and the corresponding treatment applied. Dark blue represents Zn-sufficient zones, light blue indicates Zn-deficient zones, and half/half represents heterogeneous treatments

As expected, in the homogeneous Zn-deficient medium, *NtZIP4B* promoter activity in roots increased (Fig. 2a) compared to the roots grown in homogeneous Zn-sufficient medium (Fig. 2b). This confirms that plants grown in the transparent soil medium have a similar response to Zn deficiency as those grown in hydroponic culture. However, we noticed that when plants were grown in a heterogeneous setup of the transparent soil medium (Zn-deficient and Zn-sufficient layers in one pot), lateral roots that grew in the Zn-deficient section of the medium exhibited lower *NtZIP4B* promoter activity, characteristic of Zn sufficiency (Fig. 2c, d).

To confirm this observation, we quantified the characteristic Zn-deficient tissue-specific profile of the *NtZIP4B* promoter in the epidermis and cortex (profile alpha, Fig. 2e) compared to its limited expression localization only in the central cylinder under Zn-sufficient conditions (profile beta, Fig. 2f). Profile alpha dominated in roots grown in homogeneous Zn-deficient conditions (~ 70%), while profile beta dominated in homogeneous Zn-sufficient conditions (~ 90%). Nevertheless, profile beta dominated in roots that grew in the Zn-deficient sections of the heterogeneous Zn distribution setup (both: **-Zn**/+1 µM Zn and + 1 µM Zn/**-Zn**). It appears that roots growing in the Zn-deficient part of the medium do not present a Zn deficiency response like plants grown in a homogeneously Zn-deficient medium. It is worth noting that, in this experiment, we ensured that the Zn concentration in the Zn-sufficient half of the heterogeneous treatment matched the concentration in the homogeneous Zn-sufficient medium (1 µM ZnSO₄). This means that the total Zn amount (within whole pot) in the heterogeneous treatment was half of the amount in homogeneous Zn-sufficient treatment. Despite this, roots in the Zn-deficient portion of the heterogeneous setup exhibited Zn sufficiency-like behavior.

In conclusion, these unexpected results suggest that the lack of a Zn-deficiency response in the *NtZIP4B* promoter in roots growing in the Zn-deficient region of a heterogeneously distributed Zn medium may be due to a relatively increased Zn level in those roots. This could be explained by Zn translocation from roots in Zn-sufficient areas to those in Zn-deficient regions. This finding could provide new insights into Zn homeostasis and highlight potential mechanisms of inter-root communication and nutrient redistribution that could play an important role in plant adaptation to heterogeneous environments.

### Plants appear to relocate Zn from Zn-sufficient to Zn-deficient regions

**Fig. 3:**
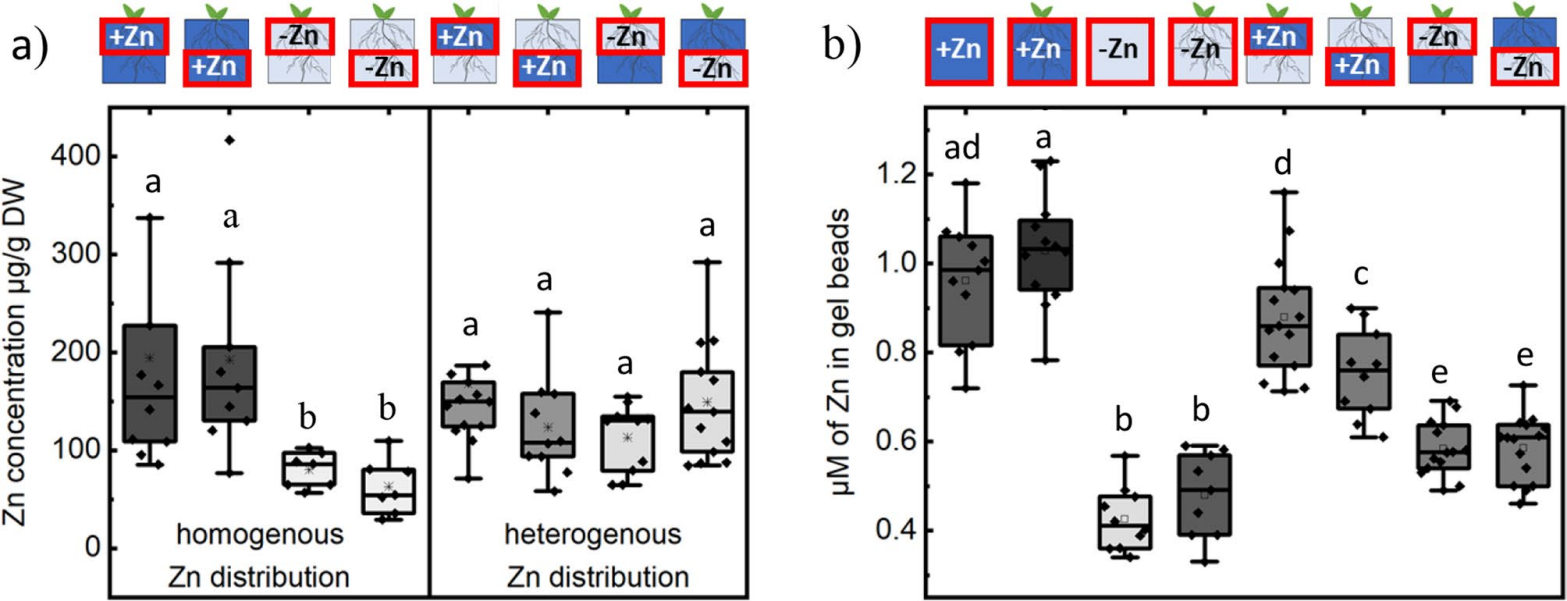
Zn concentration in plants grown in medium with heterogeneous (0/1 µM Zn; 1/0 µM Zn) Zn distribution was different from plants grown in homogeneous Zn medium (1/1 µM Zn; 0/0 µM Zn). **a** Zn accumulation in upper and lower parts of root system (µg/g DW), **(b)** Zn concentration (µM) in transparent soil before and after (14d) homogenous Zn distribution treatment and after (14d) heterogenous Zn treatment. Box plot showing data distribution with whiskers extending to the outermost points within the upper and lower inner fences (1.5 × IQR). Statistical differences (pairwise t-test) between treatments are indicated (letters denote significance, *p* < 0.05). Root system dry weight did not differ significantly. Black rhomboid shows data point. Pictograms above the graphs indicate the region of the medium where samples were taken (red rectangles) and the corresponding treatment applied. Dark blue represents Zn-sufficient zones, light blue indicates Zn-deficient zones, and half/half represents heterogeneous treatments

To further investigate whether Zn levels increase in roots growing in the Zn-deficient region of a heterogeneously distributed Zn medium, we analyzed differences in Zn concentration between roots grown under homogeneous Zn conditions (**-Zn**/**-Zn**, + 1 µM Zn/+1 µM Zn) and heterogeneous Zn conditions (**-Zn**/+1 µM Zn, + 1 µM Zn/**-Zn**), dividing the root system into halves for each treatment (Fig. 3). As expected, in homogeneous Zn conditions, Zn concentration was significantly higher in the Zn-sufficient treatment compared to the Zn-deficient treatment, regardless of whether the upper or lower part of the root system was analyzed (Fig. 3a, homogeneous Zn distribution). However, in heterogeneous Zn conditions, no significant difference in Zn concentration was observed between any of the treatments (**-Zn**/+1 µM Zn, + 1 µM Zn/**-Zn**) or between the roots growing in Zn-sufficient and Zn-deficient sections of the medium (Fig. 3a, heterogeneous Zn distribution). Interestingly, all of the samples from the heterogeneous treatments exhibited higher Zn concentrations compared to samples from homogeneous Zn-deficient treatments. There was no difference in root biomass between those plants (SI Fig. 1).

These results indicate that plants growing in media with heterogeneous Zn distribution have increased Zn level in roots that grow in Zn-deficient medium compared to plants that grow in homogenous only Zn deficient medium. We propose that this is a result of translocation of Zn within root system from roots growing in Zn sufficient to roots growing in Zn deficient medium. We also suggest that this may be due to the transport between roots rather than redistribution from stored Zn in older roots or shoots [22], as plants were grown under given conditions with only primary roots, a few short lateral roots and fraction of the shoot at the start of the experiment (SI Fig. 2). Therefore, Zn stored in the seedling is diluted during 14 days of biomass and organ growth happening directly under treatments. The Zn level in organs of plants growing in Zn deficient homogenous medium would be a representative of the maximal potential if any Zn would be redistributed from storage gained in pre-treatment growth (or seeds). We also observed that most lateral roots growing in the Zn-deficient region did not penetrate the adjacent Zn-sufficient medium, which may indicate minimal influence of Zn tropism on Zn level in root system.

As a control, we analyzed Zn concentrations in transparent soil before and after 14 days of homogeneous Zn distribution treatment, as well as after 14 days of heterogeneous Zn treatment. As expected, Zn concentrations in the transparent soil beads remained similar at the beginning and end of the experiment with homogeneous Zn distribution. This suggests that Zn-sufficient conditions remained stable for 14 days and that there was no external Zn input in Zn-deficient conditions.

Interestingly, after heterogeneous treatment, we observed a small but statistically significant decrease in Zn content in the Zn-sufficient region compared to the initial Zn-sufficient medium. It is important to remember that there is only half of Zn amount in medium with heterogenous Zn distribution compared to Zn-sufficient homogenous conditions. Therefore, this result could indicate increased Zn depletion due to increased Zn uptake by roots growing in the Zn-sufficient half, potentially to support Zn-deficient part. Similarly, we observed a small but statistically significant increase in Zn content in Zn-deficient regions compared to the initial Zn-deficient medium (Fig. 3b). This increase might be due to Zn leakage from the Zn-sufficient part. Nevertheless, in the homogeneous setup, the Zn concentration in beads placed in the top layer remained stable between the start of the experiment and after 14 days of plant growth, indicating negligible leakage from the 1 µM Zn beads. In addition, we observed similar Zn levels in the Zn-deficient beads located in both the top and bottom layers after 14 days, which further argues against leakage as a primary factor driving Zn redistribution. This is because solute movement against gravity is limited to capillary spaces, which are absent in this system, and the relatively long distance over a short time scale would constrain diffusion effects [23, 24]. A rough estimate using Fick’s second law equation (SI Eq. 1) for a finite 2D Zn source (1 µM) predicts approximately 0.084 µM Zn concentration at a distance of 2.5 cm after 14 days (an 8.4% shift from the initial value). This is likely an overestimate due to the three-dimensional structure and volume of the beads, along with restricted interconnectivity and air pockets between them, which further constrain diffusion. Instead, the increase in Zn content in beads without added Zn may indirectly support our hypothesis that Zn is translocated between lateral roots, moving from those in the Zn-sufficient region to those in the Zn-deficient region. This process could contribute to additional Zn in the local environment, possibly through root exudates. Therefore, we decided to analyze Zn distribution across the entire root system. We hypothesize that a relatively uniform Zn distribution throughout the root system of plants grown in a heterogeneous Zn medium—even in the presence of a potential Zn gradient resulting from leakage—would support the role of plant-mediated Zn translocation to lateral roots.

### Zn distribution differs in roots of plants grown in Zn heterogeneous medium

If Zn is translocated from roots with access to a Zn-sufficient medium to roots growing in Zn-deficient conditions, it is important to determine its spatial localization to identify Zn sinks in root system. However, most analytical methods used to study elemental distribution in plant samples face limitations, including low sensitivity, sample handling challenges, and pretreatment requirements that can alter metal distribution and speciation [25, 26]. Synchrotron X-ray-based techniques can overcome these limitations by providing direct, highly sensitive, spatially resolved information on the distribution of the metal and its speciation within plants, while also requiring only a limited level of sample manipulation [27, 28]. To show the Zn distribution patterns within root systems of plants grown in a medium with heterogeneous Zn distribution, we performed µXRF (micro-X-ray Fluorescence) analysis using synchrotron radiation (SOLARIS, POLYX beamline).

In the following experiments we doubled Zn concentration in one section of the container in heterogenous treatment to maintain a consistent total Zn concentration throughout the treatments. For example, by having 2 µM ZnSO_4_ in only half of the container, the average concentration across the entire volume became 1 µM ZnSO_4_, matching the control condition (1 µM Zn). We used that strategy across treatments ranging from 1 to 5 µM Zn. This ensures equivalence in Zn exposure across treatments and allows us to compare potential Zn localization.

**Fig. 4:**
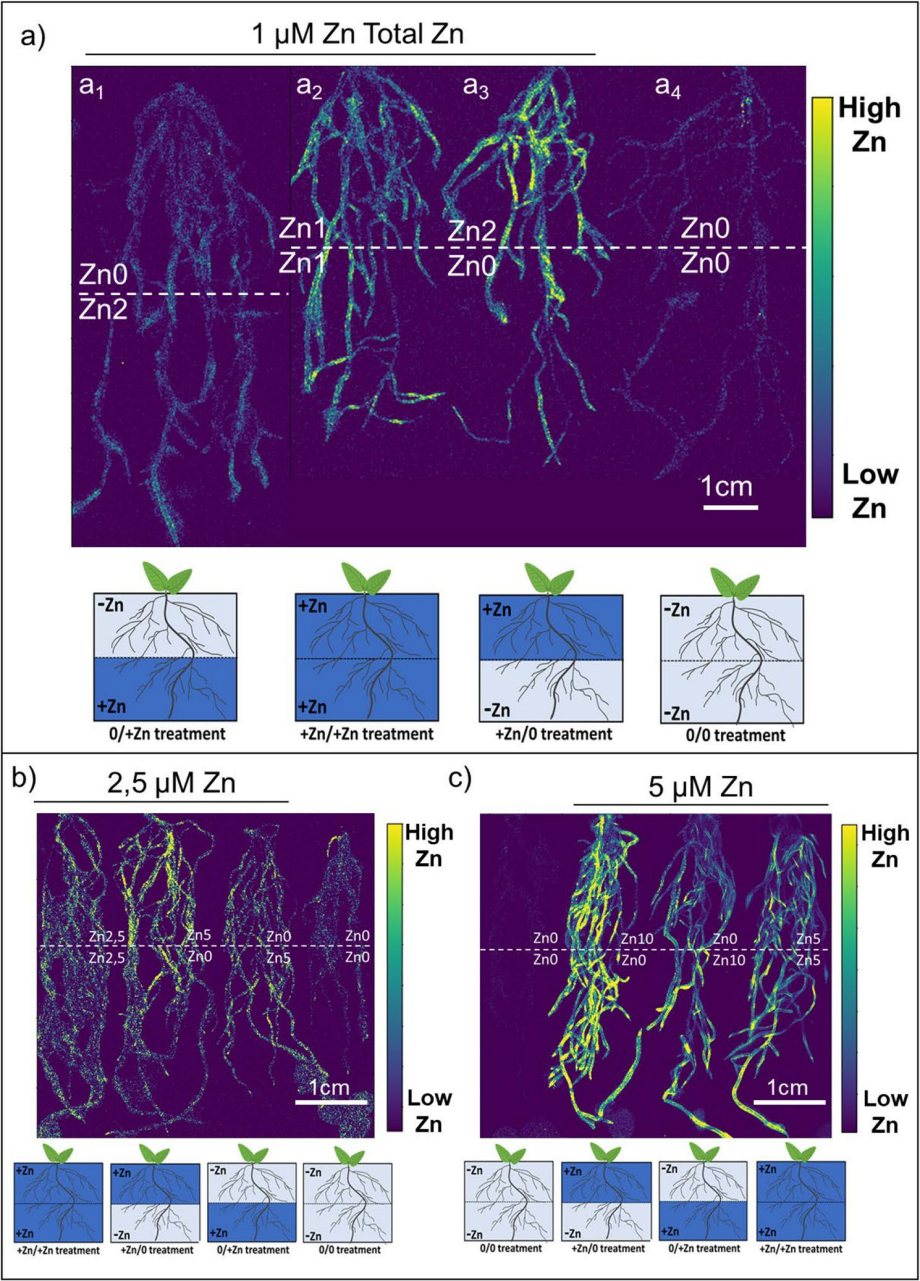
Zn is distributed from Zn-sufficient to Zn-deficient grown roots, demonstrating adaptive Zn allocation in heterogeneous Zn conditions. Visualization using Zn Kα fluorescence (8.637 keV) with a synchrotron incident beam energy of 12.5 keV at the SOLARIS POLYX beamline. **a** Representative roots (from at least 3 biological replicates) grown under heterogeneous (a1) 0/2 µM Zn and (a3) 2/0 µM Zn, and homogeneous (a2) 1/1 µM Zn and (a4) 0/0 µM Zn conditions. **b**, **c** Set of roots grown in homogenous and heterogeneous media with 2.5× and 5× higher Zn concentrations. The color bars represent normalized count rates and the lower limits of color bars were set to the background level. 3 out of 4 root systems in a) and c) were analyzed simultaneously, and other (Zn0/Zn2 and Zn5/Zn5) in consecutive run (same settings) and images were merged. Set of roots in (b) was analyzed at the same time. At least three biological replicates were analyzed per treatment, with each replicate including three root systems per beamtime. The color bar represents normalized count rates. Pictograms below the images are corresponding to the treatment applied. Dark blue represents Zn-sufficient zones, light blue indicates Zn-deficient zones, and half/half represents heterogeneous treatments

Interestingly, across all Zn treatments (ranging from 1 to 5 µM Zn), we observed a consistent pattern of Zn distribution within the root system (Fig. 4). In plants grown in homogeneous Zn-sufficient conditions, Zn was evenly distributed between the upper and lower halves of the root system (Fig. 4a–c).

As expected, in plants grown under heterogeneous Zn conditions, Zn was distributed relatively evenly (Zn0/Zn5 µM Zn and Zn0/Zn10 µM Zn have slightly increased signal in lower parts), including in roots located in the Zn-deficient part of the medium, regardless of whether the Zn-sufficient part was in the upper or lower section of the pot (Fig. 4a–c). However, an intriguing observation emerged: when Zn was localized in the upper part of the medium, its concentration in the roots appeared relatively higher compared to plants where the Zn-sufficient part was in the lower section of the pot (Fig. 4a–c). Given that the total Zn amount in these treatments was identical, this suggests that upper and lower roots may differ in their capacity for Zn uptake.

In summary, µXRF analysis corroborates that Zn could be relocated to roots growing in Zn-deficient regions. Moreover, the data indicate that Zn uptake is facilitated when increased amounts of Zn are localized in the upper part of the medium under heterogeneous Zn conditions.

### Zn localization in the medium (upper/lower) impacts Zn levels in leaves

**Fig. 5:**
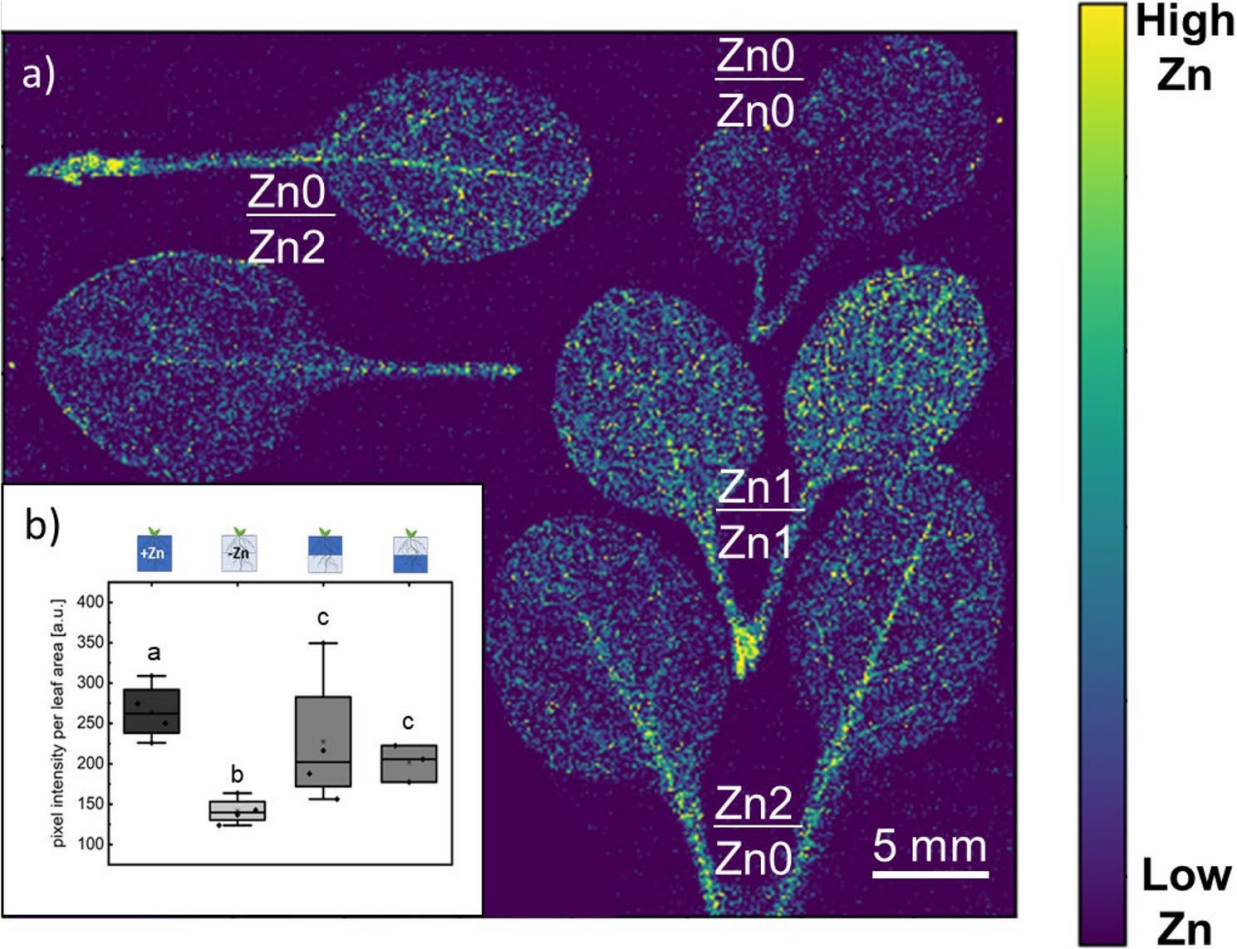
Zn translocation to leaves depends on homogenous or heterogenous Zn distribution. Visualization with Zn Kα fluorescence (8.637 keV). **a** Representative leaves (from at least 3 biological replicates) grown under heterogeneous (0/2 µM Zn; 2/0 µM Zn), and homogeneous (1/1 µM Zn; 0/0 µM Zn) conditions. Leaves were analyzed simultaneously and the colorbar represents normalized count rates and the lower limits of color bars were set to the background level. Leaves (3 replicates) were analyzed during single beamtime. **b** Comparison of relative Zn level by analysis of average pixel intensity within the leaf blade (3 replicates). Box plot showing data distribution with whiskers extending to the outermost points within the upper and lower inner fences (1.5 × IQR). Statistical differences (pairwise t-test) between treatments are indicated (letters denote significance, *p* < 0.05). Black rhomboid shows data point. Pictograms above the graph indicate the corresponding treatment applied. Dark blue represents Zn-sufficient zones, light blue indicates Zn-deficient zones, and half/half represents heterogeneous treatments

**Fig. 6:**
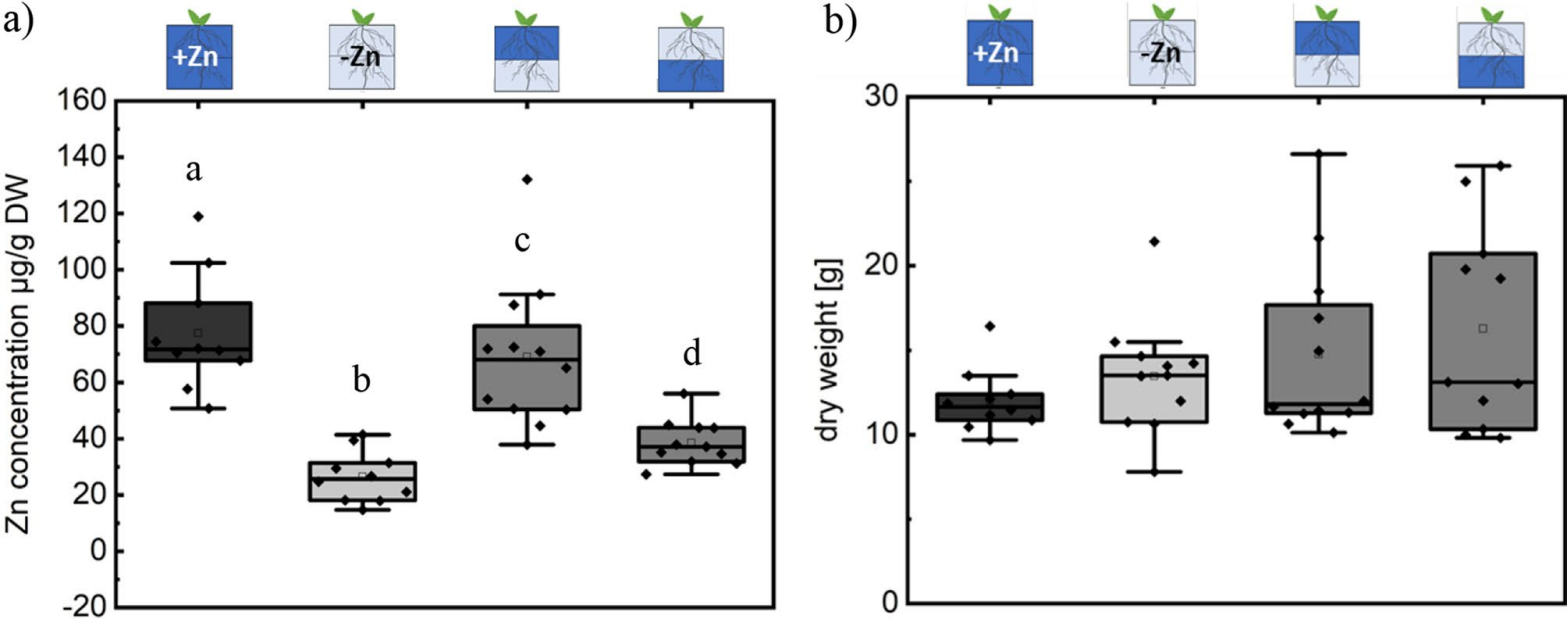
Zn concentration in shoots varies depending on whether the upper or lower root system has access to Zn. **a** Zn concentration and (**b**) dry weight of plant shoot grown in medium with homogeneous (1/1 µM Zn; 0/0 µM Zn) or heterogeneous (0/2 µM Zn; 2/0 µM Zn) Zn distribution. Box plot showing data distribution with whiskers extending to the outermost points within the upper and lower inner fences (1.5 × IQR). Statistical differences (pairwise t-test) between treatments are indicated (letters denote significance, *p* < 0.05). Black rhomboid shows data point. Pictograms above the graph indicate the corresponding treatment applied. Dark blue represents Zn-sufficient zones, light blue indicates Zn-deficient zones, and half/half represents heterogeneous treatments

Given the observed differences in Zn levels in roots grown under heterogeneous Zn localization, we mapped Zn distribution and analyzed Zn levels in leaves (excluding the hypocotyl) using µXRF. From the µXRF analysis, we found that the highest relative Zn levels were present in plants grown in homogeneous Zn-sufficient medium, while the lowest levels were observed in plants grown in homogeneous Zn-deficient medium (Fig. 5a, b). The semi-quantitative comparison of µXRF fluorescence intensities between samples (Fig. 5b) was feasible because we used leaves with specific characteristics (2 and 3 leaves) that have similar thickness, density and were analyzed in the same µXRF run. These findings were further corroborated by Zn concentration analysis in whole shoots (Fig. 6a).

In leaves from plants grown in homogeneous Zn-sufficient medium, Zn was distributed throughout the entire leaf blade (Fig. 5a). In contrast, in plants grown in medium with heterogeneous Zn distribution, Zn localization was primarily restricted to the leaf veins, accompanied by significantly lower Zn levels compared to plants grown in homogeneous Zn-sufficient medium, despite a similar total Zn content in the medium for both treatments. This indicates that heterogeneous Zn localization in the soil affects Zn distribution within the leaves.

Zn concentration analysis also revealed that shoots from plants grown in medium with heterogeneous Zn distribution exhibited lower Zn concentrations compared to those grown in the same total Zn amount under homogeneous conditions. Notably, Zn levels were higher in shoots when the Zn-sufficient medium was placed in the upper part of the pot (Fig. 6a). However, this difference in Zn distribution and concentration was not associated with significant differences in biomass, suggesting an overall lower Zn content in plants grown in medium with heterogeneously distributed Zn (Fig. 6b).

### Control over expression of Zn homeostasis related genes may play a role in Zn distribution changes in plants growing under heterogeneous Zn conditions

Finally, we analyzed the root expression of key genes involved in Zn homeostasis: *NtHMA4a/b*, encoding a major Zn exporter responsible for Zn loading into the xylem [17, 29, 30]; *NtNAS*, encoding tobacco nicotianamine (NA) synthase [31–33], which produces the Zn chelator NA involved in Zn transport in the phloem [31] and vacuolar storage [34]; and *NtZIP4B*, encoding a critical Zn transporter responsible for Zn uptake under Zn-deficient conditions [9, 33, 35]. Examining the total quantitative expression of NtZIP4B in roots also serves to complement the tissue-specific promoter localization of NtZIP4B observed in the GUS reporter assay presented in previous results.

**Fig. 7:**
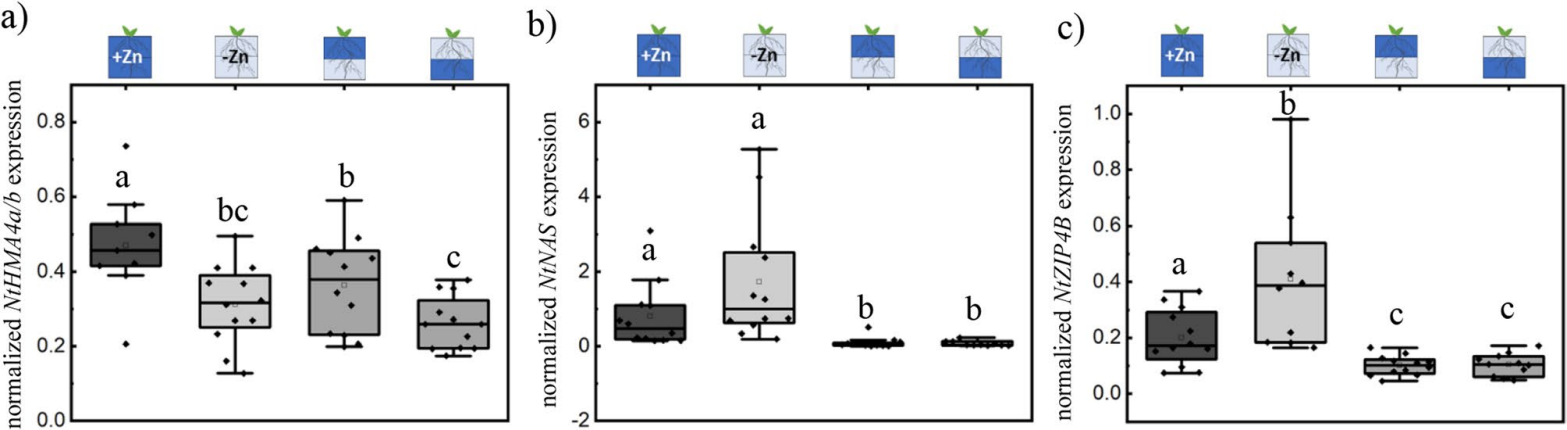
Expression of chosen Zn transporters is altered in plants grown in medium with heterogenous Zn distribution. Expression of (**a**) *NtHMA4a/b*; (**b**) *NtNAS*; c) *NtZIP4B* in roots of plants grown in medium with homogeneous (1/1 µM Zn; 0/0 µM Zn) or heterogeneous (0/2 µM Zn; 2/0 µM Zn) Zn distribution. Each root system yields 2 samples (upper and lower roots), no significant difference between those parts within each treatment were detected. Box plot showing data distribution with whiskers extending to the outermost points within the upper and lower inner fences (1.5 × IQR). Statistical differences (pairwise t-test) between treatments are indicated (letters denote significance, *p* < 0.05). Black rhomboid shows data point. Pictograms above the graph indicate the corresponding treatment applied. Dark blue represents Zn-sufficient zones, light blue indicates Zn-deficient zones, and half/half represents heterogeneous treatments

Interestingly, *NtHMA4a/b* expression was significantly reduced in roots grown in homogeneous Zn-deficient medium compared to those grown in Zn-sufficient medium. In plants grown in media with heterogeneous Zn distribution, *NtHMA4a/b* expression was similar to that observed under homogeneous Zn-deficient conditions (Fig. 7a). However, the expression of *NtHMA4a/b* varied depending on whether Zn was localized in the upper (increased expression) or lower (decreased expression) part of the medium. This pattern correlated with the Zn concentration in shoots of plants grown in heterogeneous media, suggesting a relation between NtHMA4a/b activity, Zn content in shoot and potentially Zn distribution in the medium.

The expression of *NtNAS* was similar between roots grown in homogeneous Zn-sufficient and Zn-deficient media, with a tendency for increased expression under Zn deficiency. However, in roots of plants grown in heterogeneous Zn conditions, *NtNAS* expression was significantly lower than in plants grown in Zn-sufficient homogenous medium. This reduced expression may indicate a decreased requirement for Zn chelation in plants grown in media with heterogeneous Zn distribution.

For *NtZIP4B*, the expression pattern was consistent with the promoter-GUS studies (Fig. 2), showing increased expression under Zn deficiency. However, in plants grown under heterogeneous Zn conditions, *NtZIP4B* expression was significantly lower than in plants grown in homogeneous Zn-deficient media. Similarly to *NtNAS*, the reduced expression of *NtZIP4B* in heterogeneous Zn conditions may reflect a mechanism by which plants coordinate Zn distribution to lateral roots. Earlier research from our group showed that *NtZIP4B* and *NtNAS* expression depends on Zn level in medium and increase under Zn deficiency [9, 33]. However, both *NtZIP4B* and *NtNAS* expression is suppressed in plants grown under heterogeneous Zn conditions compared to homogenous Zn-sufficient media (Fig. 7) regardless of similar Zn presence and distribution within roots (Fig. 4a). Such a result may suggest alternative regulatory mechanism for those genes that could be independent of local Zn cellular status [36] and that these mechanisms could be related to observed Zn distribution changes in plants grown in Zn heterogenous medium.

In summary, the expression of Zn homeostasis-related genes suggests that the observed Zn distribution phenotype within the root system of plants grown in a heterogeneous Zn medium may involve coordinated regulation of Zn translocation (HMA4), uptake (ZIP4B), and storage or phloem transport (NAS). Further studies are needed to reveal if those mechanisms are involved in the movement of Zn from Zn-sufficient roots to Zn-deficient roots.

## Discussion

Nutrient medium heterogeneity is known to influence plant growth and nutrient allocation, with effects varying based on plant species and root system architecture, as well as patch characteristics such as size and contrast in nutrient concentration [37]. Plants typically optimize nutrient acquisition from heterogeneous patches by altering root growth, particularly through increased lateral root elongation in nutrient-rich zones [20, 37, 38]. However, studies addressing impact of micronutrient heterogeneity in medium and how it impacts plant homeostasis, particularly Zn, are limited.

In this study, we demonstrated that a medium with heterogeneous Zn distribution, comprising alternating Zn-sufficient and Zn-deficient layers, significantly affects Zn homeostasis in plants, altering Zn distribution patterns within roots and leaves. We observed that Zn from the Zn-sufficient medium is distributed to roots growing in Zn-deficient zones. This distribution mitigates Zn deficiency responses (e.g., expression of Zn-responsive promoters) in Zn-deficient roots and limits transport of Zn to the shoot. These findings were not previously reported.

**Fig. 8:**
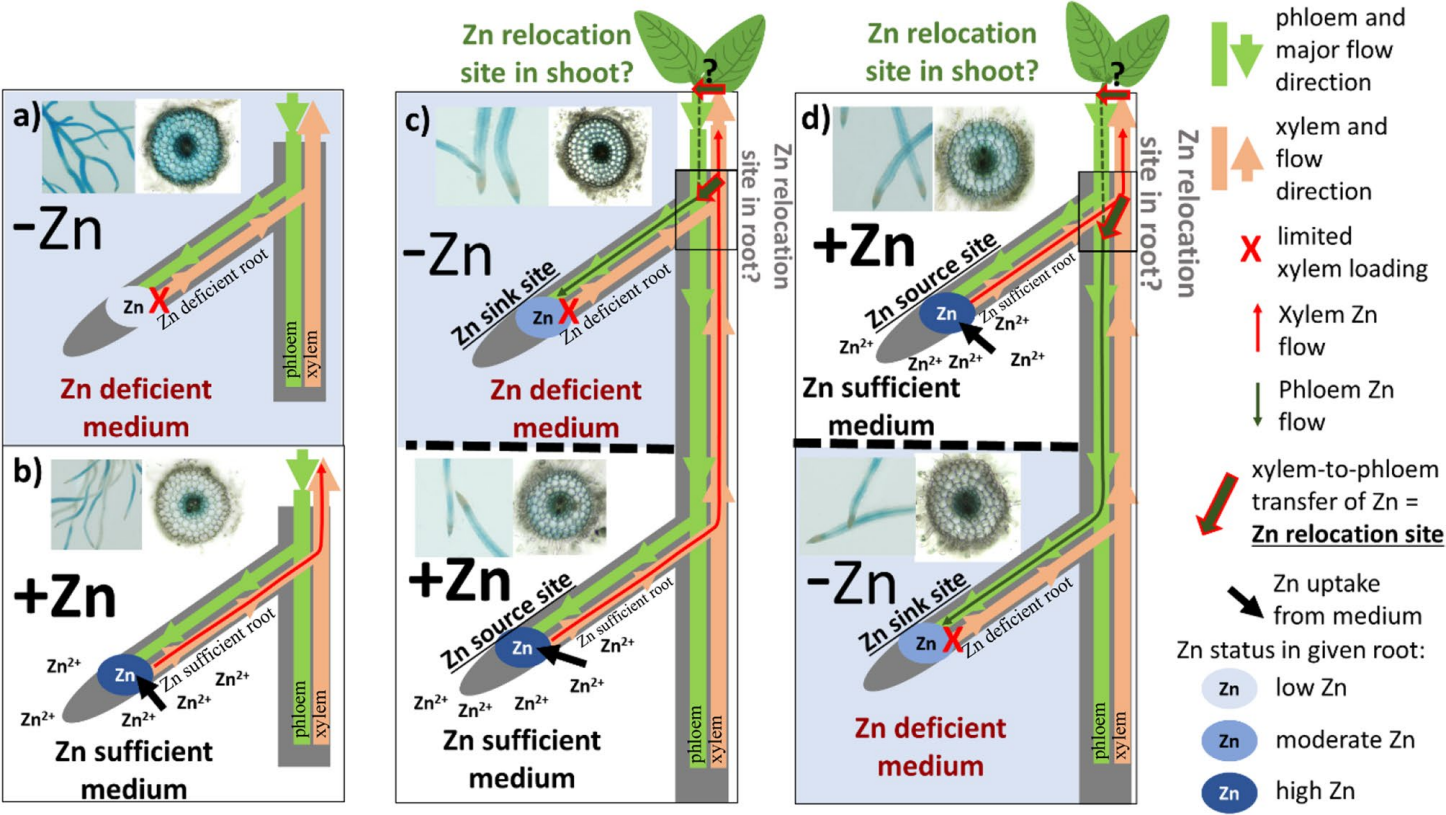
Hypothesis on how Zn^2+^ is distributed between lateral roots. **a** and (**b**) plants grown in medium with homogeneous Zn distribution, (**c**) and (**d**) plants grown in medium with heterogenous Zn distribution in upper or lower part. Under heterogeneous Zn conditions, our results suggest a potential mechanism for Zn relocation from Zn-sufficient to Zn-deficient roots. We identified hypothetical localization of these Zn transfer sites at base of root emergence zones and in the shoot (indicated by dark green arrows with red outlines). We propose that this process involves Zn unloading from the xylem, potentially mediated by Zn importers acting in xylem parenchyma cells, followed by symplastic transport of Zn or Zn-ligand complexes to the phloem. This would enable directional transport toward Zn-deficient root tips. Accordingly, we refer to these regions as xylem-to-phloem transfer sites, or Zn relocation sites

Our results suggest that Zn is transported between roots in heterogeneous Zn media. In homogeneous conditions, Zn uptake occurs across the entire root system (schematic representation in Fig. 8a, b). In heterogeneous conditions, Zn-sufficient roots act as “Zn source roots,” while Zn-deficient roots function as “Zn sink roots,” receiving Zn from Zn source roots. This redistribution does not appear to inhibit root growth, unlike deficiencies of macronutrients such as nitrogen, which are known to restrict higher-order root growth [39].

Zn translocation between roots likely involves both apoplastic (water stream) and symplastic pathways, overcoming apoplastic barriers to load Zn into the xylem, which then transports it to the shoot [40]. Given the unidirectional nature of xylem flow, this implies that nutrients may be relocated between xylem and phloem at lateral root junctions (Fig. 8c, d). We hypothesize that Zn redistribution may also occur within shoot, e.g. leaf veins, as our µXRF results show higher Zn concentrations in veins compared to the rest of the leaf blade in plants grown in heterogeneous Zn conditions. Experiments with foliar application of Zn radioisotopes (Zn^65^) in wheat and *Sedum alfredii* showed Zn translocation to leaves above and below the treated leaf, as well as to the root tips via phloem [41, 42]. The potential regulation of Zn transfer between roots could be related to a systemic signal derived from either Zn-deficient roots or shoot, similarly to proposed control over *AtMTP2* and *AtHMA2* expression in *A. thaliana* [43].

Key Zn-transporters appear to play distinct roles in mediating Zn distribution to lateral roots in heterogeneous conditions. For instance, *NtHMA4a* and *NtHMA4b*—two variants of the *HMA4* gene—may facilitate Zn loading into the xylem [30]. These gene variants exist due to the allopolyploid nature of tobacco, which originated from the hybridization of *Nicotiana sylvestris* and *Nicotiana tomentosiformis*. This genomic structure results in duplicate gene copies derived from each ancestral genome, explaining the presence of highly similar *HMA4* variants. Expression of *NtHMA4a/b* has been previously reported to be induced by Zn toxicity, but not Zn deficiency [17]. Interestingly, foliar Zn application reduced *AtHMA2* (also loads Zn to xylem) but not *AtHMA4* expression in the roots of wild-type plants grown in a Zn-deficient hydroponic solution, suggesting systemic, shoot-derived regulation of *AtHMA2* expression, but not *AtHMA4* [43]. This shows that control over Zn root-to-shoot transport is complex. In this context, it is important to notice that in our study, we used primers that detect the expression of both *NtHMA4a* and *NtHMA4b*, referred to as *NtHMA4a/b*. We observed that the expression of tobacco *HMA4a/b* was reduced in both heterogeneous Zn treatments and the homogeneous Zn-deficient treatment. Interestingly, previous studies from our group using homogeneous hydroponic conditions showed that Zn deficiency led to reduced *NtHMA4a/b* expression across the apical, middle, and basal root regions [44]. This result was accompanied by a significant (5-fold) reduction in Zn levels in roots and shoots [44]. However, in this study, Zn levels in roots did not show a dramatic difference, and slightly (less than 1-fold) lower Zn levels in shoot of plants growing in the heterogeneous Zn treatment compared to the homogeneous Zn-sufficient condition. Nevertheless, in shoots, we observed a statistically significant reduction in Zn concentration under both heterogeneous Zn conditions compared to homogeneous Zn sufficiency. Interestingly, under Zn heterogeneous conditions in medium, *NtHMA4a/b* expression was influenced by the placement of the Zn-sufficient medium part. *NtHMA4a/b* expression was higher when Zn was localized in the upper layer and lower when Zn was present in the lower layer. This could suggest that upper roots, often associated with nutrient acquisition [45], may have a greater capacity for Zn uptake and translocation to the shoot, e.g. even just by a fact that they are closer to hypocotyl.

Based on these findings, we observed a correlation between *NtHMA4a/b* expression and Zn levels in the shoots. This relationship leads us to hypothesize that the regulation of *NtHMA4a/b* expression in tobacco may be influenced by systemic shoot-derived signals especially, since there is no sequence similar to *AtHMA2* in tobacco.

Further investigation into whether the regulation of *HMA4a/b* expression plays a specific role in trafficking Zn to lateral roots is needed.

The expression of *NtNAS*, which encodes nicotianamine synthase involved in Zn binding (storage in vacuole) [34] and Zn phloem transport [46], was significantly reduced in roots grown in heterogeneous Zn media. This reduction may be needed for limiting Zn storage or phloem transport in plants that relocate Zn between their roots. We also observed a reduced Zn level in the shoots of plants grown under Zn heterogeneous conditions suggesting limited Zn translocation, which would be reasonably considered an effect of downregulation of NtNAS. In apple trees (*Malus domestica*) grown under Zn-deficient conditions, redistribution of Zn between older and younger leaves has been associated with increased expression of *MdNAS3* (nicotianamine synthase 6) and elevated nicotianamine levels in both young and mature foliage [47]. Interestingly, these studies report only a weak correlation between *NAS* expression and nicotianamine concentrations in corresponding tissues, suggesting the involvement of additional regulatory mechanisms. It is plausible that other metal-binding ligands may contribute to Zn relocation under deficiency, pointing to a more complex network than currently understood. In this context, our findings—showing reduced *NtNAS* expression and presumably lower nicotianamine levels—may reflect diminished capacity for Zn loading into the phloem. We propose that Zn redistribution may occur within a spatially restricted root region, likely where lateral roots emerge. Specific cell populations in this area may provide localized conditions conducive to Zn translocation, including enhanced synthesis of ligands required for effective phloem transport. This will be a subject of our further investigation.

Finally, *NtZIP4B*, a Zn uptake transporter that was activated under Zn-deficient conditions, was suppressed in heterogeneous Zn media, despite the presence of Zn-deficiency. In our study, we examined the expression pattern of *pNtZIP4B::GUS* under both Zn heterogeneous and Zn-sufficient homogeneous conditions. We observed that its expression was limited to the cortex and central cylinder. It is possible that reduced Zn transport to cell in central cylinder could impact Zn apoplastic/symplastic balance which itself is known to regulate Zn homeostasis [48, 49]. This would also facilitate apoplastic transport and potentially xylem loading, reducing Zn retention in Zn-sufficient roots. This however do not explain how Zn would be redirected to Zn-deficient lateral roots as the same mechanisms would reduce potential for phloem loading in part of the root where Zn have to be transferred from xylem to phloem (Fig. 8d). This might suggest that Zn relocation to Zn-deficient parts that should involve Zn phloem transport would involve xylem-to-phloem reloading in shoot. We plan to investigate that in our future studies.

Previous studies on Arabidopsis and tobacco show that Zn transport and homeostasis are tightly regulated by collective functioning of different Zn transporters/chelators like HMA, NAS and ZIP family transporters [9, 18, 29, 30, 33, 35, 48, 50–52]. The process by which Zn-sufficient roots in heterogeneous media provide Zn to Zn-deficient roots likely involves a complex Zn transport mechanism that requires coordinated regulation. This process to function efficiently is probably limited to specific part of root or shoot where xylem-phloem Zn relocation occurs (Fig. 8d). Interestingly, both *NtZIP4B* and *NtNAS* promoters have Zinc Deficiency Response Element (ZDRE) motif [9], regulated by bZIP19 and bZIP23 transcription factors which induce expression of these genes under Zn cellular deficiency. This mechanism would allow to alter expression of genes between Zn-sufficient and Zn-deficient parts of the root system [9, 53]. We also anticipate that some kind of signaling between different root levels, or shoot-to-root, may be involved in Zn partitioning within the root system (Fig. 8d). We also anticipate the involvement of additional proteins in Zn relocation and its regulation. However, further studies on signaling, systemic responses and cellular mechanisms that facilitate Zn-dependent control over Zn supply are needed.

## Materials and methods

### Plant material, growth conditions, and treatments

The experiments were conducted using tobacco (*Nicotiana tabacum* var. Xanthi) plants. Seeds originating from stock obtained in 2002 from the Institute of Biochemistry and Biophysics PAS, Warsaw, Poland and have since been propagated in the greenhouse of the University of Warsaw for experimental purposes.

Plants were grown in a controlled environment chamber under the following conditions: 23/16°C day/night temperature, 40–50% relative humidity, 16 h photoperiod, and photosynthetically active radiation (PAR) of 250 µmol m⁻² s⁻¹ provided by fluorescent Flora tubes.

Seeds were surface sterilized using 8% (w/v) sodium hypochlorite for 2 min, rinsed thoroughly, and germinated on vertically positioned Petri dishes containing quarter-strength Knop’s medium supplemented with 2% (w/v) sucrose and 1% (w/v) agar. After three weeks of growth (SI Fig. 2), seedlings were transferred to transparent soil medium in 7 × 10 cm Magenta^®^ boxes and grown for an additional two weeks.

### Preparation of transparent soil

Transparent soil was prepared by creating gel beads through the dropwise addition of a sodium alginate: Phytogel^®^ (Sigma) mixture (1:4) into a 10 mM MgCl₂ solution, which immediately polymerized the outer layer to form spherical beads, as described previously [21]. A sterile, custom-made system was used to facilitate the production of large quantities of transparent soil (Fig. 1, SI Fig. 3).

The bead diameter was controlled by the size of the tip opening; beads approximately 0.5 cm in diameter were used in this study. Beads were crosslinked for 4 h, rinsed with deionized sterile water, and then transferred to an equal volume of liquid half-strength Knop’s medium with adjusted Zn concentration: 0, 2, 4, 5, 10 and 20 µM ZnSO₄ that after diffusion would result with ¼ Knop’s medium containing 1, 2, 2,5, 5, or 10 µM ZnSO₄ accordingly. We also prepared medium without added Zn. Beads were left in the medium overnight to allow full nutrient diffusion.

For experimental setups, two layers of beads (~ 100 g each) were placed in Magenta^®^ boxes, with layer boundaries marked on the box side to identify root sections exposed to each treatment. Residual fluid accumulating at the bottom was removed with a sterile pipette. Seedlings were positioned in holes made with a pipette tip to ensure root growth through both medium layers.

### GUS assay

Plants expressing *promNtZIP4B::GUS* and wild-type (WT) plants (as staining controls) [9]were fixed in 90% ice-cold acetone for 25 min with gentle rotation. Samples were washed four times in reaction buffer (50 mM Na₂HPO₄, pH 7.0, and 0.2% Triton X-100), with the third wash performed under mild vacuum (0.04 bar).

Samples were then transferred to reaction buffer containing 2 mM X-Gluc (5-bromo-4-chloro-3-indolyl β-d-glucuronic acid), infiltrated under vacuum for 15 min, and incubated at 37 °C in the dark for 2.5 h with gentle shaking. Samples were cleared in ethanol solutions of increasing concentration (50%, then 75%) before scanning the entire root system (EPSON V850 Pro).

To visualize *NtZIP4B* expression at the tissue/cellular level, stained root fragments were embedded in 3% agarose and sectioned at 50–150 μm thickness using a vibratome (Leica VT1000S). Sections were analyzed microscopically (OPTA-TECH microscope). Differences in staining intensity were quantified using ImageJ. Based on the observations, Zn-deficient (alpha) and Zn-sufficient (beta) *NtZIP4B* expression patterns were identified (Fig. 2e, f). The frequency of expression pattern was determined by analyzing main and all lateral roots apical segment (up to 2 cm above root tip) from scans of entire root systems. Data came from 3 independent experiments (biological) with at least 14 plants for each heterogenous treatment at least 9 plants for each homogenous setup. The sample number depends on root penetration into the + Zn/**-Zn** treatment layers in the heterogeneous setup, and approximate depth matching those layers in the homogeneous setup.

### Determination of metal concentrations

At the end of the transparent soil experiment, upper and lower root sections were separated. Shoots were rinsed briefly with deionized water. Roots were washed sequentially with Milli-Q water, 5 mM CaCl₂ (4 °C, 15 min under agitation) to remove unbound and weakly bound metals from the apoplast, and finally with water.

Samples were dried at 55 °C, and dry biomass was measured. Digestion was performed using 65% HNO₃ and 39% H₂O₂ (9:1, v: v) in a closed microwave mineralizer (Milestone Ethos 900, Milestone, Bergamo, Italy) [30]. Zn concentrations were measured using flame atomic absorption spectrophotometry (AAS; TJA Solution Solar M, Thermo Electron Manufacturer Ltd, Cambridge, UK). Certified reference material (Virginia tobacco leaves, CTA-VTL-2) was included in each analysis. The plants were cultivated in two independent experiments: the first included six plants per treatment, and the second included seven. Zn concentration was assessed from at least 7 plants. These were selected from a group of 12 plants, based on root penetration into the + Zn/**-Zn** treatment layers in the heterogeneous setup, and approximate depth matching those layers in the homogeneous setup.

### µXRF analysis

Micro-XRF 2D mapping experiments were carried out at the PolyX beamline [54] of the SOLARIS National Synchrotron Radiation Centre [55]. Monochromatic beam from SOLARIS bending magnet (1.3T) was generated with a Mo/B_4_C multilayer monochromator with 1.3% bandwidth. The beam was focused with an ellipsoidal monocapillary optics (Sigray, 20 nm thick Pt inner coating, 20 mm working distance) to focal spot of 5µm. Samples were placed on a system of translation stages to perform 2D scans. Spectra were acquired with two Hitachi Vortex EM360 silicon drift detectors (ML3.3 extreme and 25 µm thick Be windows, 0.5 mm thick Si sensor, active area 100mm^2^) coupled to XGlab Dante digital pulse processor. Detectors were placed in backscattering geometry (45 degree from the sample surface) and the incident X-ray beam was normal to the sample surface. 2D Maps were acquired using Mapping mode of the Dante DPP with a fast continuous horizontal and vertical point-by-point motions. In 2D Maps the pixel size was 100 µm (one instance of 50 µm for Zn5/Zn5 image) with dwell time 12.5 ms per single spectrum. To create the 2D maps, count-rates in energetic region-of-interests were normalized by detector’ live time and incident beam intensity measured with ionization chamber. Data from two detectors were summed up taking into account the ratio between their response to the Zn signal.

Samples of root systems and leaves (2nd and 3rd) were exercised, rinsed with deionized water, and dried using paper towels. Samples were mounted in a 3D-printed polymer holder with a 5 × 5 cm mounting area. Roots were sandwiched between 3.6 μm foil (Spectro-Film™, DuPont) and gel beads (approx. 0,5 cm diameter containing ¼ Knop medium without Zn) were added was added on sides to prevent desiccation during analysis. At least three biological replicates were analyzed per treatment, with each replicate including three root systems per beamtime. Leaves (3 replicates) were analyzed during single beamtime.

### RNA extraction

Total RNA was extracted from samples stored at −80 °C using the Universal RNA Purification Kit (EURx, #E3598) according to the manufacturer’s instructions. RNA concentration and purity were assessed using a NanoDrop ND-1000 spectrophotometer (Nanodrop, Wilmington, DE, USA), with 260/280 ratios ranging between 1.8 and 2.0. RNA integrity was verified by agarose gel electrophoresis. The plants were cultivated in two independent experiments, with six plants per treatment. RNA was isolated from at least nine plants. These were selected from a group of twelve plants, based on root penetration into the + Zn/**-Zn** treatment layers in the heterogeneous setup, and approximate depth matching those layers in the homogeneous setup. The roots were divided into two (upper and lower) samples before RNA extraction and without pooling. There was no significant differences in expression between upper and lower sample.

### Quantitative real-time PCR

cDNA was synthesized from total RNA using the RevertAid™ First Strand cDNA Synthesis Kit (Thermo Fisher Scientific) following the manufacturer’s protocol. RT-qPCR was conducted using the LightCycler^®^ 480 System (Roche) and SYBR Green Master Mix (Roche, #0488735001).

Primers (Supplementary Table 1) were designed using IDT OligoAnalyzer and OligoCalc tools. The reference gene *NtPP2A* (protein phosphatase 2 A; AJ007496) was co-amplified with the target genes to normalize expression levels. Reactions were performed in triplicate for each independent biological replicate. Relative transcript levels were calculated using the ΔCt method.

The quality of qPCR results was assessed using amplification and melting curves, with non-template controls included in each assay. Primers are listed in SI.

### Data presentation and statistical analysis

We used a box plots to show data distribution with whiskers extending to the outermost points within the upper and lower inner fences (1.5 × IQR). Significant differences between treatments was measured using pairwise t-tests (*p* < 0.05) in Excel with Real Statistics Resource Pack (Release 8.9.1; Copyright 2013–2023; Charles Zaiontz. www.real-statistics.com). The µXRF maps images were prepared using developed phyton based tool (code in supplementary information) from original data saved as.mat files and converted to.tiff format.

## Conclusion

Our study demonstrates that plants grown in media with heterogeneous Zn distribution exhibit a unique mechanism for Zn allocation, providing the first evidence that Zn may be delivered from Zn-sufficient roots to Zn-deficient roots within the same root system. This finding highlights the potential for studying previously unknown Zn homeostasis mechanisms in roots, driven by dynamic and intricate processes likely involving Zn transporters.

Additionally, our results indicate that Zn distribution within the root system limits Zn translocation to the shoot. Zn is important for proper cell division in root tip meristematic tissue [56]. By temporarily limiting Zn levels in the shoot, the plant may prioritize root growth, increasing its potential to outgrow Zn-deficient areas. This adaptation ultimately enhances survival in a heterogeneous soil environment. We propose that Zn distribution to lateral roots involves the downregulation of Zn-deficiency-related genes and the movement of Zn to Zn-deficient regions, potentially mediated by xylem-phloem interactions and nicotianamine transport. Uncovering these mechanisms, which appear to operate independently of external Zn concentrations, will offer new opportunities to manipulate Zn distribution within plants to enhance nutrient use efficiency and adaptability to suboptimal growth conditions.

Our findings offer valuable insights into the complex regulation of Zn homeostasis and open new avenues for exploration. Future studies should investigate specific sites of Zn relocation in both roots and shoots, complemented by tissue-specific expression analyses of known Zn transporters. Additionally, exploratory RNA-seq approaches may help identify previously unknown genes encoding proteins involved in Zn distribution. The role of phloem transporters, Zn content in phloem sap, and the molecular mechanisms governing Zn sensing, signaling, and allocation in heterogeneous environments are also key areas for deeper investigation.

## Supplementary Information

Supplementary Material 1.

## Data Availability

Data are available from the corresponding author upon reasonable request.

## Abbreviations

CAX: Cation/proton eXchanger
CDF/MTP: Cation Diffusion Facilitator/Metal Tolerance Protein
GUS: ß-glucuronidase
HMA: Heavy Metal ATPase
NA: nicotianamine
NAS: nicotianamine synthase
PAR: photosynthetically active radiation
VIT: Vacuolar Iron Transporters
WT: wild-type
YSL: Yellow Stripe-Like
ZDRE: Zinc Deficiency Response Element
ZIF: Zinc-Induced Facilitator
ZIP: ZRT/IRT-like proteins
