# Supplementary material for: Zinc translocation from Zn-sufficient to Zn-deficient roots as an adaptation to heterogeneous Zn availability
Source: BMC Plant Biol. 2025 Oct 8;25:1341. doi: 10.1186/s12870-025-07391-z (PMC12506409; doi:10.1186/s12870-025-07391-z)
Supplement: Supplementary file 1 — Supplementary Material 1. [file 12870_2025_7391_MOESM1_ESM.docx]

Supplementary data

**Zinc Translocation from Zn-Sufficient to Zn-Deficient Roots as an Adaptation to Heterogeneous Zn Availability**

Magdalena Pypka**^1^**, Diana Davydenko **^1^**, Katarzyna Sowa**^2^**, Julia Maksymiuk**^1^**, Paweł Wróbel**^2,4^**, Tomasz Kołodziej**^2^**, Paweł Korecki**^3^** and Oskar Siemianowski**^1^***

**^1^**University of Warsaw, Faculty of Biology, Institute of Experimental Plant Biology and Biotechnology, Department of Plant Metal Homeostasis, Miecznikowa Street 1,
02-096, Warsaw, Poland

**^2^**National Synchrotron Radiation Centre SOLARIS, Jagiellonian University,

Czerwone Maki 98; 30-392 Krakow, Poland

^3^Institute of Physics, Jagiellonian University, Łojasiewicza 11, 30-348 Krakow, Poland

^4^AGH University of Krakow, Faculty of Physics and Applied Computer Science, al. Mickiewicza 30, 30-059 Krakow, Poland

^*^corresponding author: o.siemianowski@uw.edu.pl


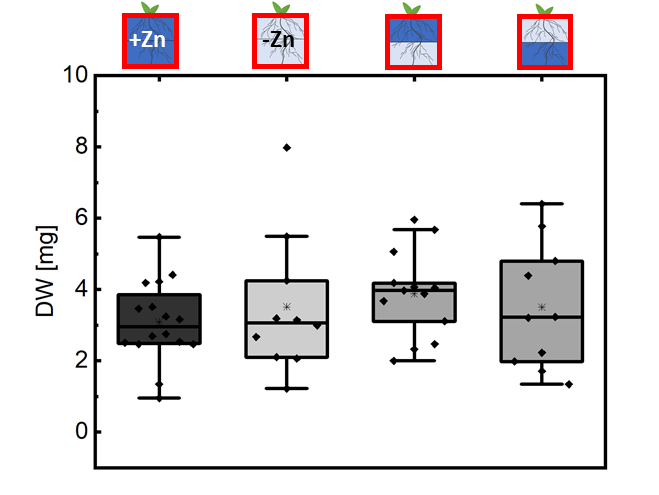


**a)**

**SI Fig 1.** Root dry weight from plants grown in medium with homogeneous (1/1 µM Zn; 0/0 µM Zn both a) and b)) or heterogeneous Zn distribution (sum of upper and lower part): a) 0/1 µM Zn; 1/0 µM Zn and b) 0/2 µM Zn; 2/0 µM Zn. Box plot showing data distribution with whiskers extending to the outermost points within the upper and lower inner fences (1.5 × IQR). Significant differences between treatments are indicated by pairwise
t-tests (p < 0.05). Black rhomboid shows data point. Pictograms above the graph indicate the region of the medium where samples were taken (red rectangles) and the corresponding treatment applied. Dark blue represents Zn-sufficient zones, light blue indicates Zn-deficient zones, and half/half represents heterogeneous treatments.


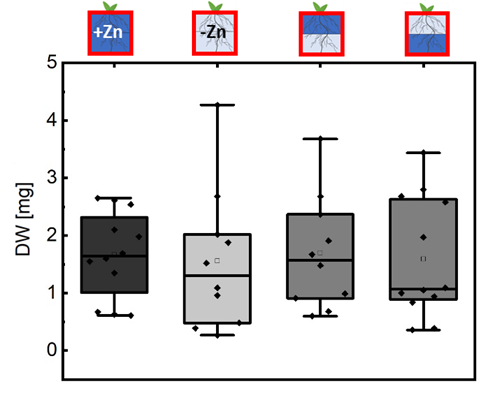


**b)**


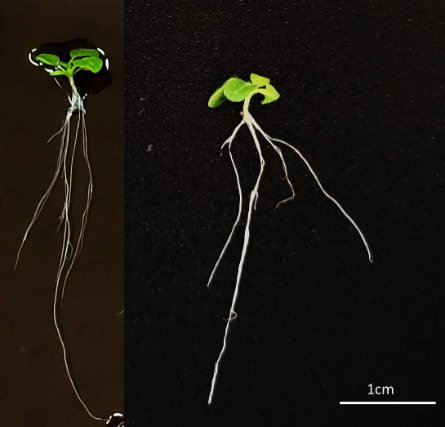


**SI Fig 2.** 3-week old plant, before transfer to transparent soil.


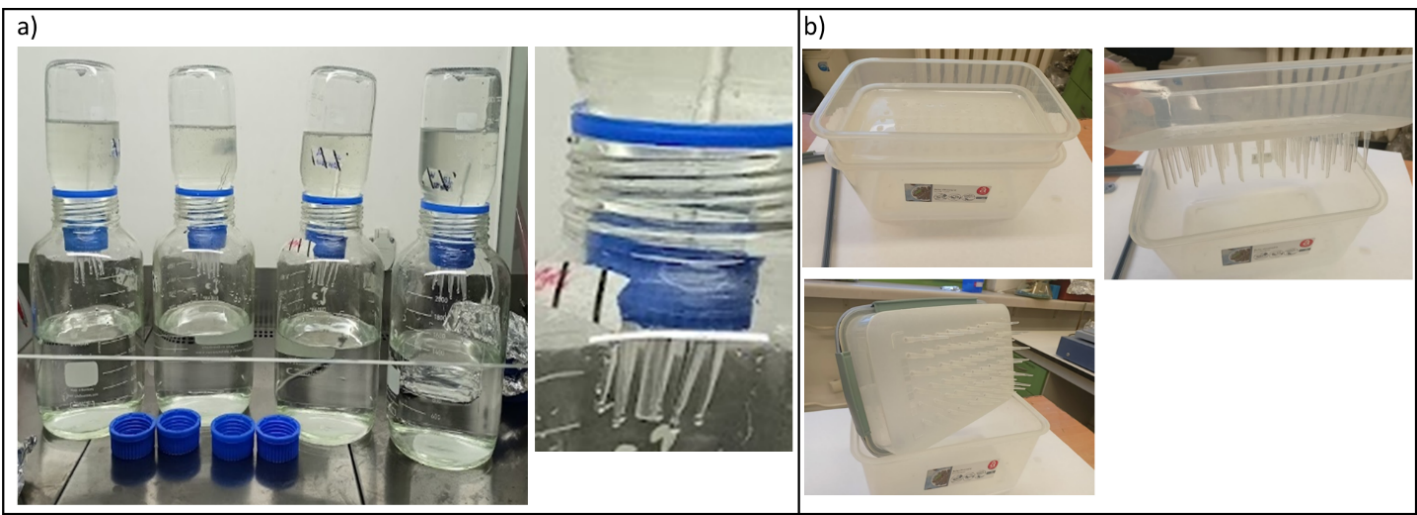


**SI Fig 3.** Sterile high-throughput production of transparent soil: a) using sterile 400ml GL40 bottle with transparent soil polymer mix, in 2L GL80 bottle with 1,2 L crosslinking solution. Screwcap was drilled and up to eight 200μL cut tips with was inserted. The Pauster glass pipet was added for facilitated air intake. b) commercial (Auchan) polypropylene (heat resistant) set made of flat and tall box that fit into each other. Flat box is drilled (up to 60 holes) and 200 μL cut tips are inserted. The choice of system depends on size of autoclave in which system need to be sterilized.

SI Table 1

Primers:

***NtZIP4B*** *- XM_016586154.1*

ZIP4B-Forward: TCTGTTTCCAATATTACCTGC

ZIP4B-Revers: TTCTTGCCAACTAACGGG

***NtHMA4*** *– both HMAa* (HF937053.1) *and HMAb* (HF675180.1)

NtHMA4-Forward: TCTGTTTCCAATATTACCTGC

NtHMA4 -Revers: TTCTTGCCAACTAACGGG

***NtNAS*** - both tobacco NAS: NAS1 (XM_016633252.1); NAS2 (NM_001326005.1)

NtNAS-Forward: ATCTCGTCTCGTGGCATC

NtNAS-Reverse: TCCTTATCCATACCAACCAACG

Equation 1: Fick's Second Law (Finite 2D Solution):

$$\boldsymbol{C}\left( \boldsymbol{x, t} \right)\boldsymbol{=}\frac{\boldsymbol{C}_{\boldsymbol{0}}}{\left( \boldsymbol{2}\sqrt{\left( \boldsymbol{\pi D t} \right)} \right)}\boldsymbol{*}\mathbf{ex}\mathbf{p} \left( \boldsymbol{-}\frac{\boldsymbol{x}^{\boldsymbol{2}}}{\left( \boldsymbol{4 D t} \right)} \right)$$

Where:

- C(x, t) is the concentration at position x and time t
- C_0_ is the initial concentration or scaling factor
- D is the diffusion coefficient – we used 1.918*10^-6^ [1] shown for Zn diffusion in agarose 1.5% gel.
- t is time
- x is the spatial distance from the origin

**Code for μXRF map figures:**

import os

from PyQt5.QtWidgets import (

QApplication, QMainWindow, QFileDialog, QPushButton, QLabel, QVBoxLayout, QWidget, QLineEdit, QListWidget, QInputDialog

)

from PyQt5.QtCore import Qt

import matplotlib.pyplot as plt

from matplotlib.widgets import RectangleSelector

from PIL import Image

import numpy as np

import tifffile as tiff

from scipy.ndimage import rotate

class ImageProcessor(QMainWindow):

def __init__(self):

super().__init__()

self.initUI()

self.selected_folder = None

self.files = []

self.current_file = None

self.img_data = None

self.roi = None

self.linear_selector = None

def initUI(self):

self.setWindowTitle('TIFF Image Processor')

self.setGeometry(100, 100, 800, 600)

layout = QVBoxLayout()

# Folder selection

self.folder_btn = QPushButton('Select Folder')

self.folder_btn.clicked.connect(self.select_folder)

layout.addWidget(self.folder_btn)

# Element filter

self.filter_label = QLabel('Enter Element Abbreviation (e.g., Zn, Fe):')

layout.addWidget(self.filter_label)

self.filter_input = QLineEdit()

layout.addWidget(self.filter_input)

# File list

self.file_list = QListWidget()

self.file_list.itemClicked.connect(self.display_image)

layout.addWidget(self.file_list)

# Rotation buttons

self.rotate_90_btn = QPushButton('Rotate 90°')

self.rotate_90_btn.clicked.connect(lambda: self.rotate_image(90))

layout.addWidget(self.rotate_90_btn)

self.rotate_180_btn = QPushButton('Rotate 180°')

self.rotate_180_btn.clicked.connect(lambda: self.rotate_image(180))

layout.addWidget(self.rotate_180_btn)

self.rotate_270_btn = QPushButton('Rotate 270°')

self.rotate_270_btn.clicked.connect(lambda: self.rotate_image(270))

layout.addWidget(self.rotate_270_btn)

# Custom rotation button

self.rotate_custom_btn = QPushButton('Rotate by Custom Angle')

self.rotate_custom_btn.clicked.connect(self.custom_rotate_dialog)

layout.addWidget(self.rotate_custom_btn)

# Save button

self.save_btn = QPushButton('Save Image')

self.save_btn.clicked.connect(self.save_image)

layout.addWidget(self.save_btn)

# Main widget

container = QWidget()

container.setLayout(layout)

self.setCentralWidget(container)

def select_folder(self):

self.selected_folder = QFileDialog.getExistingDirectory(self, "Select Folder")

if self.selected_folder:

self.filter_files()

def filter_files(self):

self.file_list.clear()

element_filter = self.filter_input.text()

if self.selected_folder:

self.files = [

os.path.join(root, file)

for root, _, files in os.walk(self.selected_folder)

for file in files

if file.endswith('.tiff') and element_filter in file

]

self.file_list.addItems(self.files)

def display_image(self, item):

self.current_file = item.text()

try:

# Read the image

self.img_data = tiff.imread(self.current_file)

# If the image has multiple channels, take the first channel

if self.img_data.ndim > 2:

self.img_data = self.img_data[:, :, 0]

self.show_image()

except Exception as e:

print(f"Error reading file: {e}")

def show_image(self):

"""Display the current image with options for ROI selection."""

fig, ax = plt.subplots()

im = ax.imshow(self.img_data, cmap='viridis')

plt.colorbar(im, ax=ax)

# Add Rectangle ROI Selector

self.roi = RectangleSelector(ax, self.onselect_rectangle, drawtype='box', useblit=True, button=[1], interactive=True)

plt.show()

def onselect_rectangle(self, eclick, erelease):

"""Handle rectangle ROI selection."""

x1, y1 = int(eclick.xdata), int(eclick.ydata)

x2, y2 = int(erelease.xdata), int(erelease.ydata)

roi_data = self.img_data[y1:y2, x1:x2]

mean_intensity = np.mean(roi_data)

vertical_profile = np.mean(roi_data, axis=1)

horizontal_profile = np.mean(roi_data, axis=0)

print(f"Rectangle ROI Mean Intensity: {mean_intensity}")

plt.figure()

plt.subplot(1, 2, 1)

plt.plot(horizontal_profile)

plt.title('Horizontal Profile')

plt.subplot(1, 2, 2)

plt.plot(vertical_profile)

plt.title('Vertical Profile')

plt.show()

def rotate_image(self, angle):

"""Rotate the image by the given angle."""

if self.img_data is not None:

self.img_data = rotate(self.img_data, angle, reshape=False, mode='nearest')

self.show_image()

def custom_rotate_dialog(self):

"""Prompt user to enter a custom rotation angle."""

angle, ok = QInputDialog.getDouble(self, "Rotate Image", "Enter Rotation Angle (in degrees):", 0, -360, 360, 1)

if ok:

self.rotate_image(angle)

def save_image(self):

if self.img_data is not None:

save_path, _ = QFileDialog.getSaveFileName(self, "Save Image", filter="TIFF Files (*.tiff);;JPEG Files (*.jpg)")

if save_path:

Image.fromarray(self.img_data).save(save_path)

print(f"Image saved to {save_path}")

if __name__ == '__main__':

app = QApplication([])

window = ImageProcessor()

window.show()

app.exec_()

1. Patil S, Adhyapak N: **OBSTRUCTION EFFECT AND SELF-DIFFUSION OF ZINC ION IN AGAR-GEL**. *INDIAN JOURNAL OF CHEMISTRY SECTION A-INORGANIC BIO-INORGANIC PHYSICAL THEORETICAL & ANALYTICAL CHEMISTRY* 1981, **20**(11):1079-1081.
